# Placebo Use in the United Kingdom: Results from a National Survey of Primary Care Practitioners

**DOI:** 10.1371/journal.pone.0058247

**Published:** 2013-03-20

**Authors:** Jeremy Howick, Felicity L. Bishop, Carl Heneghan, Jane Wolstenholme, Sarah Stevens, F. D. Richard Hobbs, George Lewith

**Affiliations:** 1 Department of Primary Care Health Sciences, University of Oxford, Oxford, United Kingdom; 2 Centre for Applications of Health Psychology, Faculty of Social and Human Sciences, University of Southampton, Southampton, United Kingdom; 3 Health Economics Research Centre, Department of Public Health, University of Oxford, Old Road Campus, Headington, Oxford, United Kingdom; 4 Primary Care and Population Sciences, Faculty of Medicine, University of Southampton, Aldermoor Health Centre, Southampton, United Kingdom; University of Louisville, United States of America

## Abstract

**Objectives:**

Surveys in various countries suggest 17% to 80% of doctors prescribe ‘placebos’ in routine practice, but prevalence of placebo use in UK primary care is unknown.

**Methods:**

We administered a web-based questionnaire to a representative sample of UK general practitioners. Following surveys conducted in other countries we divided placebos into ‘pure’ and ‘impure’. ‘Impure’ placebos are interventions with clear efficacy for certain conditions but are prescribed for ailments where their efficacy is unknown, such as antibiotics for suspected viral infections. ‘Pure’ placebos are interventions such as sugar pills or saline injections without direct pharmacologically active ingredients for the condition being treated. We initiated the survey in April 2012. Two reminders were sent and electronic data collection closed after 4 weeks.

**Results:**

We surveyed 1715 general practitioners and 783 (46%) completed our questionnaire. Our respondents were similar to those of all registered UK doctors suggesting our results are generalizable. 12% (95% CI 10 to 15) of respondents used pure placebos while 97% (95% CI 96 to 98) used impure placebos at least once in their career. 1% of respondents used pure placebos, and 77% (95% CI 74 to 79) used impure placebos at least once per week. Most (66% for pure, 84% for impure) respondents stated placebos were ethical in some circumstances.

**Conclusion and implications:**

Placebo use is common in primary care but questions remain about their benefits, harms, costs, and whether they can be delivered ethically. Further research is required to investigate ethically acceptable and cost-effective placebo interventions.

## Introduction

Surveys in various countries suggest 17% to 80% of doctors have prescribed ‘placebos’ in routine clinical practice, [1], [2], [3], [4], [5], [6], [7], [8], [9], [10], [11], [12] yet placebo use outside the context of a clinical trial with full informed consent is generally considered unethical. [13], [14], [15], [16] The only survey of placebo use in the UK was a 1976 qualitative study restricted to Welsh practitioners. [2] Hence current prevalence of placebo use in UK primary care is unknown.

A barrier to investigating placebo use is that confusion surrounds the ‘placebo’ concept. [17], [18], [19], [20], [21], [22] For example, placebos are often characterized as inactive and nonspecific when in fact they can be active, and have specific effects, especially for relieving pain. [23], [24], [25], [26], [27] Since this was an empirical rather than conceptual study we adopted a pragmatic approach and asked doctors whether they used various treatments described as placebos in other similar surveys. Our approach has the advantage of being useful: patients, doctors, and policy makers care more about whether particular treatments are effective and ethical than whether these treatments carry the label ‘placebo’.

We aimed to discover if UK general practitioners (GPs) prescribe placebos as frequently as elsewhere and also to understand the conditions under which general practitioners find placebos ethical. [13], [14] Ethical placebos may have a role in health care, for example in treating patients with chronic osteoarthritic pain, where current best practice often involves medications such as non-steroidal anti-inflammatory drugs (NSAIDs) that have known harmful side effects. [28], [29], [30]Our aim was not to study placebo use within the context of controlled trials.

## Methods

### Participants

Participants in this cross-sectional survey were randomly sampled from among the UK general practitioners registered with a clinician marketing service (Doctors.net); 71% of UK GPs are registered with the service. Ethical approval was obtained from the University of Oxford, and the survey was sent via email on 26 April 2012. Email reminders were sent out on 30 April and 4 May, and the survey closed on 21 May 2012.

We required 655 responses for our sample to reflect the population with 99% confidence (±5%). Based on recent surveys, [4], [11] we predicted a response rate of between 40 to 60% and emailed the survey to 1,715 general practitioners.

### Defining pure and impure placebos

Following other recent surveys we adopted the convention of dividing placebos into ‘pure’ and ‘impure’. [4], [7], [10], [11], [12] Pure placebos are interventions such as sugar pills (which are available commercially [31]) or saline injections without direct pharmacologically active ingredients for the condition being treated. Impure placebos are substances, interventions or ‘therapeutic’ methods which have known pharmacological, clinical or physical value for some ailments but lack specific therapeutic effects or value for the condition for which they have been prescribed. These may include:

- Positive suggestions
- Nutritional supplements for conditions unlikely to benefit from this therapy (such as vitamin C for cancer)
- Probiotics for diarrhea
- Peppermint pills for pharyngitis
- Antibiotics for suspected viral infections [5]
- Sub-clinical doses of otherwise effective therapies [32]
- Off-label uses of potentially effective therapies
- Complementary and Alternative medicine (CAM) whose effectiveness is not evidence-based [33], [34]
- Conventional medicine whose effectiveness is not evidence-based [35], [36], [37], [38]
- Diagnostic practices based on the patient's request or to calm the patient such as
  - Non-essential physical examinations
  - Non-essential technical examinations of the patient (blood tests, X-rays)

### Survey instrument

To compare our results with other surveys of placebo use we adapted recently published questionnaires for a UK audience. [4], [7], [9], [10], [11], [12] We piloted our questionnaire with GP colleagues at Oxford and Southampton (*n* = 21) to ensure face validity. The questionnaire asked respondents to note how frequently (if at all) they used placebo interventions. Additional items asked about reasons for placebo use, circumstances under which practitioners felt placebo use was ethically acceptable, and what practitioners told patients when they prescribed placebo interventions. To address the risk of social desirability bias in our responses our questionnaire began with two case studies that avoided using the term ‘placebo’, one about using antibiotics for throat infections and the other about a hospital patient who responded when the intravenous painkillers were replaced by saline injection. To minimize conceptual ambiguity we included our definitions of pure and impure placebos on the first and subsequent pages of the questionnaire, and respondents were offered the option to answer that a given intervention was not a placebo. Our questionnaire included open-ended questions where respondents could provide comments about their definitions of placebos.

### Statistical analysis

Participants entered their responses directly into an online survey using Confirmit. [39] We used descriptive statistics (means and 95% confidence intervals) to describe practitioner characteristics and frequencies of placebo use. We reported how often respondents used all pure and all impure placebos at least once in their career (mean and 95% CI). For each type of placebo we categorized prevalence of use into: frequent (daily or approximately once per week), occasional (approximately once per month or once per year) and rare/never (more than once per year or never). We also noted reasons for prescribing placebos (mean and 95% CI), and attitudes towards the ethics of placebos. We analyzed differences (RR and 95% CI) between usage among respondents who stated placebos were categorically unacceptable in routine practice and those who stated placebos were sometimes acceptable.

We used Fisher's exact test to investigate whether placebo use was associated with gender, year of qualification (stratifying 1989 or earlier/1990–1999/2000 or later), number of patients treated per week (100 or less/101 to 150/151 or more), number of days per week in current practice (0–3/3.5–4.5/5 or more). A Bonferroni correction was used to allow for the 14 types of placebo against which each characteristic (gender, year qualified, etc.) was tested: each test was deemed significant if p<0.0036, giving a 5% type I error rate across all 14 tests for each characteristic. All analyses were conducted using STATA (version 11).

## Results

### Respondent characteristics

Of the 1715 primary care practitioners sent the questionnaire 783 (46%) responded. There were more male (55%) respondents, the average year of qualification was 1993 (range 1964 to 2007, mode = 2000), the average days per week in current practice were 4 (range 0.5 to 6), and the mean number of patients treated per week was 123 (range 6 to 450). Our participants were similar to those registered with the General Medical Council (GMC): 52% of UK general practitioners are male, and the mode of UK GP qualification year is 2000. [40], [41], [42] One respondent reported treating zero patients per week and working clinically zero days per week and we excluded them from the analysis.

### Pure placebos: prevalence of use

12% (95% CI 10 to 15) of respondents reported using pure placebos (sugar pills or saline injections) at least once in their career (see Table 1). 1% (95% CI 0 to 2) of respondents reported using pure placebos at least once per week (see Table 2).

**Table 1:** Summary of placebo usage.

	Frequency (percentage, 95% CI)	
	Has used at least once in career	Has never used/is not a placebo
**Pure placebos**	12% (9.0 to 14.6)	88% (85.4 to 90.0)
**Impure placebos**	97% (96.0 to 98.6)	3% (1.7 to 4.0)

**Table 2:** Frequency of placebo use by type of placebo.

	Frequency (percentage, 95% CI)			This is not a placebo
	Frequently (daily or approximately once per week)	Occasionally (approximately once per month or at least once in the last year)	Rare (less than once per year or never)	
**PURE PLACEBOS**	0.90% (0.2 to 1.6)	1.5% (0.7 to 2.4)	97.4% (96.3 to 98.5)	0.3% (0.0 to 0.6)
Sugar Pills	0.5% (0 to 1.0)	1.0% (0.3 to 1.7)	97.8% (96.8 to 98.8)	0.6% (0.1 to 1.2)
Saline injections	0.4% (0 to 0.8)	1.4% (0.6 to 2.2)	97.6% (96.5 to 98.6)	0.6% (0.1 to 1.2)
**IMPURE PLACEBOS**	77.0% (74.0 to 79.9)	18.0% (15.3 to 20.7)	4.6% (3.1 to 6.1)	0.4% (0.0 to 0.8)
Positive suggestions	51.7% (48.2 to 55.2)	19.6% (16.8 to 22.3)	18.3% (15.6 to 21.0)	10.5% (8.3 to 12.6)
Nutritional supplements	5.9% (4.2 to 7.5)	23.9% (20.9 to 26.9)	68.8% (65.6 to 72.0)	1.4% (0.6 to 2.2)
Probiotics for diarrhea	9.0% (7.0 to 11.0)	39.0% (35.6 to 42.4)	45.5% (42.0 to 49.0)	6.5% (4.8 to 8.3)
Peppermint pills for pharyngitis	1.8% (0.9 to 2.7)	6.5% (4.8 to 8.3)	89.8% (87.6 to 91.9)	1.9% (1.0 to 2.9)
Antibiotics for suspected viral infections	25.2% (22.2 to 28.2)	51.2% (47.6 to 54.7)	19.7% (16.9 to 22.5)	4.0% (2.6 to 5.3)
Sub-clinical doses of effective therapies	4.9% (3.6 to 6.4)	34.4% (31.1 to 37.7)	57.3% (53.8 to 60.8)	3.5% (2.2 to 4.7)
Off-label uses of a potentially effective therapy	13.0% (10.7 to 15.4)	45.4% (41.9 to 48.9)	33.6% (30.3 to 36.9)	7.9% (6.0 to 9.8)
Complementary and Alternative medicine (CAM) whose effectiveness is not evidence-based	6.8% (5.0 to 8.5)	44.2% (40.8 to 47.7)	44.8% (41.3 to 48.2)	4.2% (2.8 to 5.6)
Conventional medicine whose effectiveness is not evidence-based	26.2% (23.1 to 29.3)	51.2% (47.6 to 54.7)	16.0% (13.4 to 18.6)	6.6% (4.9 to 8.4)
Non-essential physical examinations	53.6% (50.1 to 57.1)	28.6% (25.5 to 31.8)	12.4% (10.1 to 14.7)	5.4% (3.8 to 7.0)
Non-essential technical examinations of the patient (blood tests, X-rays)	31.2% (28.0 to 34.4)	50.5% (47.0 to 54.0)	13.6% (11.2 to 16.0)	4.7% (3.2 to 6.2)
OTHER*	36.4% (33.0 to 39.7)	27.3% (24.3 to 30.4)	18.2% (15.5 to 20.9)	18.2% (15.5 to 20.9)

* CBT, ferrous sulphate, gesture and intonation in addition to positive suggestion, medication, physiotherapy, joint injection, reassurance, referral to website, reassurance, ‘tell them my own or my family member with the same problem’, unnecessary referrals.

Reasons for prescribing pure placebos varied. 55% (95% CI 51 to 59) of respondents reported prescribing pure placebos to induce possible psychological treatment effects, 33% (95% CI 30 to 36) to calm patients, 32% (95% CI 29 to 35) because the patient requested a therapy, and 31% (95% CI 28 to 34) to treat non-specific complaints.

### Impure placebos: prevalence of use

97% (95% CI 96% to 98) of respondents reported using impure placebos at least once in their career (see Table 1), and 77% (95% CI 74 to 79) reported using impure placebos frequently (at least once per week, see Table 2). Several impure placebos were used frequently by at least a quarter of GPs. These included non-essential physical examinations (54%, 95% CI 50 to 57), positive suggestions (52%, 95% CI 48 to 55), non-essential technical examinations (31%, 95% CI 28 to 34), conventional medicine whose effectiveness is not evidence-based (26%, 95% CI 23 to 29), and antibiotics for suspected viral infections (25%, 95% CI 22 to 28).

Common reasons for prescribing impure placebos were similar to reasons for prescribing pure placebos. 50% (95% CI 47 to 54) reported prescribing them for a possible psychological treatment effect, 45% (95% CI 42 to 49) because the patient requested a therapy, 35% (95% CI 32 to 39) for non-specific complaints, and 32% (95% CI 29 to 35) to calm patients.

### Pure placebos: ethical attitudes

66% (95% CI 63 to 70) of respondents felt there were circumstances in which pure placebos were ethically acceptable (see Table 3). Yet 82% (95% CI 79 to 85) stated pure placebos were unacceptable when they involved deception, and 90% (95% CI 88 to 92) stated they were unethical when they endangered patient/doctor trust. Half (53%) of doctors who prescribed pure placebos told patients that ‘this therapy has helped many other patients,’ a quarter (25%) told patients that the treatment promoted self-healing and a tenth (9%) told the patient the treatment was a placebo. Doctors who reported finding pure placebos ‘never acceptable in clinical practice’ were less likely to prescribe them (6% versus 15%, relative rate (RR) 0.41, 95% CI 0.25 to 0.69). Respondents who reported finding pure placebos ‘never acceptable’ prescribed them for possible psychological treatment effect or to offer treatment to those with untreatable/incurable disease. Sample sizes were too small to formally analyze reasons for placebo use among doctors who found pure placebos never ethically acceptable.

**Table 3:** Summary of practitioner beliefs about ethical acceptability of placebo use.

	Percentage (95% CI) of GPs agreeing with statement for	
Statement	PURE placebos	IMPURE Placebos
Placebos **are** acceptable when used for their psychological effect	52.8% (49.3 to 56.3)	58.3% (54.9 to 61.8)
Placebos **are** acceptable when all other therapies have been exhausted	49.7% (46.2 to 53.2)	66.5% (63.2 to 69.8)
Placebos **are** acceptable when the patient wants or expects this therapy	47.2% (43.7 to 50.7)	46.7% (43.2 to 50.2)
Placebos **are** acceptable when clinical experience has shown a benefit	60.7% (57.3 to 64.2)	79.0% (76.2 to 81.9)
Placebos **are not** acceptable when they involve deception	82.0% (79.3 to 84.7)	82.4% (79.7 to 85.0)
Placebos **are not** acceptable when they endanger patient/doctor trust	90.2% (88.1 to 92.2)	93.6% (91.9 to 95.3)
Placebos **are not** acceptable because the efficacy is insufficient	52.8% (49.3 to 56.3)	35.0% (31.7 to 38.4)
Placebos **are not** acceptable because of legal problems	61.4% (58.0 to 64.8)	45.3% (41.8 to 48.8)
Placebos **are not** acceptable because they have possible adverse effects	41.6% (38.1 to 45.0)	55.8% (52.3 to 59.2)
Placebos **are** acceptable in some circumstances in clinical practice	66.2% (62.9 to 69.6)	84.1% (81.6 to 86.7)
Placebos **are never** acceptable in clinical practice	32.5% (29.2 to 35.8)	13.9% (11.5 to 16.4)

### Impure placebos: ethical attitudes

84% (95% CI 82 to 87) of respondents agreed there were some circumstances in which impure placebos were ethically accepable (see Table 3). Yet 82% (95% CI 80 to 85) stated impure placebos were unacceptable when they involved deception and 94% (95% CI 92 to 95) stated they were unethical when they endangered patient/doctor trust. Of doctors who prescribed impure placebos, half (48%) told patients ‘this therapy has helped many other patients,’ 18% told patients that the treatment promoted self-healing, and 8% told the patient the treatment was a placebo. Three respondents who used the free text option noted saline injections were useful for treating opiate addicts inappropriately presenting to emergency departments. Doctors who reported finding impure placebos ‘never acceptable in clinical practice’ are also less likely to prescribe them in clinical practice’ (91% versus 99%, RR 0.92, 95% CI 0.86 to 0.98). GPs who prescribed impure placebos in spite of finding them ‘never acceptable’, did so for the following common reasons: to calm the patient, because the patient requested treatment, for possible psychological treatment effect or as a supplement to medication. Sample sizes were too small to formally analyze reasons for placebo use among doctors who never found impure placebo use to be ethically acceptable.

### Predictors of placebo use

There were differences between males and females in the frequency of use of several placebos. More females used positive suggestions on a frequent basis (64% versus 52%, RR 1.23, 95% CI 1.08 to 1.40, *P* = 0.0013), and more males prescribed off-label uses of potentially effective therapy frequently (18% versus 10% RR 1.82, 95% CI 1.23 to 2.71, *P* = 0.0029). There was a significant association between the number days per week spent in practice and the frequency of use of non-essential physical exams. 92% of those working 0–3 days in practice used non-essential physical exams frequently or occasionally compared to 77% of those working 5 days or more (RR 1.19, 95% CI 1.07 to 1.32, *P* = 0.0014). We suspected females spent fewer days in practice and this might have confounded the association between sex and frequency of non-essential examinations. We repeated Fisher's exact test and found no significant associations between the number of days per week spent in practice and frequency of non-essential physical exam use for males or females (*P* = 0.142).

## Discussion

### Summary of main findings

Placebos may represent one of the more commonly used treatments in UK primary care in spite of (perhaps sometimes unjustified) ethical constraints. Twelve percent of respondents reported using pure placebos, and 97% reported using impure placebos at least once in their career. Many placebos were used frequently by over half the respondents, and most general practitioners felt there were circumstances in which impure and pure placebos were ethically acceptable. Half of the practitioners who use placebos informed their patients that this intervention has helped other patients without specifically telling them that they were prescribing a placebo. This raises unresolved ethical issues about how GPs approach informed consent in relation to their prescriptions of placebos. The analysis indicates potential gender differences in the frequency of placebo prescriptions.

### Strengths and Limitations

Given ethical constraints surrounding placebo use in clinical practice, general practitioners completing surveys may have understated their use of placebos. The response rate (46%) raises questions about representativeness, yet our respondents were similar to GPs registered with the GMC.

Our pragmatic definition of ‘placebo’, while consistent with other surveys, could be challenged. For example, it might turn out that some conventional therapies that lack an evidence base (considered to be placebos in our survey) are eventually proven to be efficacious non-placebo treatments. In addition the distinction between pure and impure placebos is only useful as a rough guide. Just as antibiotic treatments can function as treatments (for bacterial infections) or placebos (for viral infections), so sugar pills and saline solutions can be treatments for some conditions and placebos for others. For instance sugar is not inert with respect to diabetes. [18], [22], [43] and saline solution is an effective treatment to treat increased intracranial pressure. [44] The difference between pure and impure placebos is therefore stochastic: pure placebos are less often used as treatments as compared with impure placebos.

We considered that for practical and internationally comparative purposes it is more important to describe treatments often labeled as placebos rather than resolve the philosophical debate over the definition of placebos. UK GPs appeared to agree with our definition. 82% – 99% (depending on which placebo treatment) agreed that the treatments listed as placebos were, in fact, placebos.

Other limitations include potential recall and response bias. [45], [46] Given the ethical strictures against placebo use, these biases may have let to an underestimate of reported placebo usage. [47]

### Costs and side-effects

A full cost analysis of placebo prescriptions is beyond the scope of this work yet our data suggest placebos probably cost the NHS many millions of pounds each year. [48], [49] Besides the possible financial burden, placebos can be harmful (in which case they are referred to as ‘nocebos’ [50], [51]). For example impure placebos such as antibiotics can have serious adverse (‘nocebo’) effects. [52]

### Other literature

The survey instrument was derived from previously published investigations and enables our data to be easily compared with other international studies. A 2009 systematic review of 22 surveys of placebo use in general practice in 12 countries found 17% to 80% of practitioners had used ‘pure’ placebos at least once in their career and between 54% and 57% had used impure placebos at least once in their career. [5] The latest survey of placebo use was published after the systematic review and found 45% of German GPs had used pure placebos and 76% had used impure placebos in the last year. [12] Hence the results of our UK survey are internationally consistent.

### Implications for future research and clinical practice

Placebos are commonly used in UK primary care. Clinical and health service researchers have spent decades investigating ways to effectively utilize ‘active’ conventional treatments safely, ethically and intelligently. [53] The time has come to use similar methods to investigate ways to rationalize placebo use. The long term viability of placebo use in clinical practice depends on whether placebo benefits outweigh harms, [54] their cost, and whether patients and practitioners deem their use to be ethically acceptable. Further investigations are warranted to develop ethical and cost-effective placebos.

### Ethical approval

Ethical approval was granted by the University of Oxford.

### Data sharing

The questionnaire, and dataset are available from the corresponding author (jeremy.howick@phc.ox.ac.uk).
